# The Association Captured

**Published:** 1886-03

**Authors:** 


### "THE ASSOCIATION CAPTURED," AND LED ASTRAY.

"Dr. D. W. Yandell made his first appearance as a delegate from Kentucky in 1856, then in '57 and '59. His name does not appear again [in the transactions] (nor his State) till in 1871, when, in San Francisco, he was elected President. Neither his State nor he had any claims to the honor. It was, so report said, due to designing, ambitious, selfish and industrious plotting that the Presidency was awarded him. Since that time * * [he] has attended only four meetings, and since 1874, *none at all.* [Eleven years] With the exception of his address as President, no other contribution appears from his pen to the Association. In his address he says 'The Association is making our profession one in heart throughout our borders."—*Truth, in Journal A. M. A.*

Now the doctor says "disappointed office seekers" "captured" the A. M. A., and at New Orleans "led it astray;" and forsooth, he will help "bust it up."

Reading the above, inspired our Dr. Merryman's muse, and he handed us the following:

